# A population-based cohort of young women diagnosed with breast cancer in Geneva, Switzerland

**DOI:** 10.1371/journal.pone.0222136

**Published:** 2019-09-06

**Authors:** Robin Schaffar, Christine Bouchardy, Pierre Olivier Chappuis, Alexandre Bodmer, Simone Benhamou, Elisabetta Rapiti

**Affiliations:** 1 Geneva Cancer Registry, Global Health Institute, University of Geneva, Geneva, Switzerland; 2 Service of Oncology, Geneva University Hospitals, Geneva, Switzerland; 3 Service of Genetic Medicine, Geneva University Hospitals, Geneva, Switzerland; University of Kansas Medical Center and VA Medical Center, UNITED STATES

## Abstract

**Purpose:**

Breast cancer is the most frequently diagnosed cancer among women worldwide. Despite the fact that breast cancer is more frequent after fifty years of age, breast cancer among young women has recently drawn particular attention due to an increase in incidence in several western countries. With the exception of individuals with a high genetic risk, breast cancer occurring in younger women remains poorly understood. This project aims at investigating the patient, tumour and treatment characteristics as well as the long-term health outcomes of these women by evaluating numerous variables that were collected from their pathology and medical files, including the social environment, family history, fertility and pregnancy.

**Participants:**

We constituted a population-based cohort from the Geneva Cancer Registry of 1586 patients with breast cancer who were aged less than 46 years at the time of diagnosis.

**Findings to date:**

Breast cancer was diagnosed before the age of 35 years in 225 women (14.2%), between 35 and 39 years of age in 368 women (23.2%) and between 40 and 45 years of age in 993 women (62.6%). Most of the patients were diagnosed with luminal A or luminal B molecular subtypes (32.8 and 37.5%, respectively), stage I or II tumours (75.2%), and estrogen (74.8%) and progesterone (67.5%) positive receptors. During the study period, 16.7% of these women developed loco-regional recurrences and 25.4% developed distant metastases; the majority (66.3%) did not have a recurrence. Regarding mortality, 474 (29.9%) women died during the study period, 347 (73.2%) from breast cancer.

**Future plans:**

The results of this study will help filling the knowledge gap about treatment of young breast cancer patients and having a child after breast cancer, and will provide clinicians and public health professionals’ with additional information to improve quality of care and decrease the impact of breast cancer in young women.

## Introduction

Breast cancer is by far the most frequently diagnosed cancer among women around the world, with about 1.7 million new cases annually and almost half of them in countries with a high level of development [1]. Although breast cancer is more frequent in women above fifty years of age, recently breast cancer in young women has drawn particular interest as several studies have found increasing incidence rates in women less than 40 years old [2,3]. In this age group there is a higher proportion of patients with pathogenic variants in genes that predispose to breast cancer, such as *BRCA1* or *BRCA2*, compared with patients who have onset of breast cancer at an older age [4]. Because of their young age, these women are not invited to breast cancer screening programs and are therefore more likely to be diagnosed with symptomatic disease or an advanced stage. Several studies have described worse clinical outcomes and more long-term treatment complications among these young women as compared to older breast cancer patients [5–13]. In particular, women aged less than 40 years have a higher risk of loco-regional recurrence compared to older patients and an increased risk of distant metastasis and death [14]. However, a large meta-analysis of 66 studies could not conclude whether this worse prognosis is associated with *BRCA* mutations [15]. Recently, no effect of *BRCA1*/*BRCA2* mutations on the overall survival or distant disease-free survival of early breast cancer patients was found in a large prospective cohort in the United Kingdom [16]. Identifying factors that contribute to loco-regional recurrence-free survival and distant recurrence-free survival can therefore help clinicians to take appropriate decisions about the management of these patients.

In developed countries, approximately 13% of breast cancer diagnoses occur during the reproductive years (20–44 years) and about 2% of women are diagnosed before 35 years of age [17]. The current trend of delayed childbearing in many developed countries may increase the risk of breast cancer during pregnancy or in the first 12 months postpartum (pregnancy-associated breast cancer). In addition, as many young breast cancer survivors may not have constituted their families, the decision on whether and how to become pregnant after treatment is of great importance to these women, their families, and their physicians. The recommendations that are available for counseling this population on the safety of carrying a pregnancy during pregnancy-associated breast cancer or becoming pregnant after treatment for breast cancer are not definitive as they are mainly based on observational studies and systematic reviews [18–20].

The proposed population-based project aims to evaluate tumour specifics, patterns of care (including genetic counselling), prognostic factors, and outcomes (including loco-regional, distant recurrence, second cancer and death) of breast cancer among women aged 45 years or less. After establishing the prevalence of pregnancies among these breast cancer patients, the second aim is to evaluate characteristics, treatment, and prognosis of breast cancer diagnosed during pregnancy, shortly after (notably during breastfeeding) and/or substantially after in young women.

## Cohort description

### Study population and study participants

Between 1970 and 2012, 1,586 women in the Geneva Cancer Registry database were identified as resident in the canton of Geneva and diagnosed with a primary invasive non-metastatic (M0) breast cancer at the age of 45 years or less. The study population included women aged up to 45 years of age to increase the sample size, pregnancy events and other outcomes. This also allows a comparison of young patients (40–45 years old) and very young patients (<40 years old). Patients with a previous malignant cancer and/or *in situ* breast cancer that occurred before or within 6 months of the breast cancer diagnosis were excluded from the cohort. No selection was made regarding a woman’s *BRCA1/BRCA2* status or other genetic predispositions to breast cancer.

### Data routinely collected by the Geneva Cancer Registry

The Geneva Cancer Registry, created in 1970, was the first cancer registry in Switzerland and one of the oldest in Europe. It is one of the few registries in the world that registers an extended set of clinical data, particularly on the method of diagnosis (including screening), stage, treatment, survival, and second tumours (all tumours are exhaustively recorded). It records information on all incident cases of malignant neoplasms occurring in the population of the canton (approximately 490,000 inhabitants). The Registry collects information from various sources and has high accuracy as evidenced by the very low percentage (<2%) of cases recorded only from death certificates [21]. Data collection is based on a voluntary agreement between the recording medical institutions of the canton of Geneva and the Registry. All hospitals, pathological laboratories and practitioners report information on all current and past cancer cases. Data are systematically abstracted from hospital and laboratory records by trained registrars. To collect missing clinical and therapeutic data, questionnaires are sent out regularly to the private practitioners. In addition to the official underlying cause of death derived from the death certificate, registrars use all available information to establish, where relevant, a revised underlying cause of death.

Formal ethical approval and patient consent for this study was not required as the Geneva Cancer Registry has a general authorization to collect nominative data and analyse anonymized data. This authorization was given in 1991 by the Federal Expert Commission for data protection and research (https://unige.ch/medecine/rgt/questcequunregistredestumeurs/).

#### Additional data collection from medical charts

All patient medical records were retrospectively reviewed to collect additional information or to complete missing data on the patient, tumour, and treatment characteristics, and on health outcomes including loco-regional and distant recurrences using a structured and validated questionnaire. When no information on recurrence was found in the clinical files the patients were considered as relapse-free.

#### Additional data collection from genetic counselling

Data from the Oncogenetics and Cancer Prevention Unit of the Oncology Service at the Geneva University Hospitals was merged with the Geneva Cancer Registry data to identify women who underwent counselling and were tested for *BRCA1* or *BRCA2* germline pathogenic variants.

#### Additional data collection from the Cantonal Population Office

The Cantonal Population Office (OCP) is a regional administration office that monitors births, deaths, migration, residency and civil partnerships. The Geneva Cancer Registry database was merged with that of the OCP to identify and collect information on the offspring (including stillbirths) who were born within 12 months before and 9 months after breast cancer diagnosis among the 1,586 breast cancer patients. This linkage was done using a unique personal identification number registered in both databases and was used to cross-validate information on children who were born before or after the breast cancer diagnosis as collected from the medical records. All of the variables in the database are described in Table 1.

**Table 1:** List of variables.

**Data collected by the Geneva Cancer Registry**			
**Patient characteristics**		**Diagnostic circumstances**	
▪	Age	▪	Method of diagnosis
▪	Gender	▪	Presence of symptoms
▪	Nationality	▪	Methods of assessment
▪	Place of birth	**Sector of care**	
▪	Marital status	**Treatment**	
▪	Last occupation	▪	Type of surgery
**Tumour characteristics**		▪	Radiotherapy
▪	Histological type	▪	Chemotherapy
▪	Grade (since 1990)	▪	Endocrine therapy
▪	Estrogen receptor status (since 1995)	▪	Surgical margin (since 2010)
▪	Progesterone receptor status (since 1995)	**Follow-up**	
▪	HER2 overexpression status (since 2003)	▪	Survival status
▪	Size of the tumour	▪	Cause of death
▪	Clinical TNM	**Others**	
▪	Pathological TNM	▪	Subsequent tumour occurrence
▪	Sentinel lymph node biopsy	▪	Family history of breast and/or
▪	Number of lymph nodes resected		ovary cancer (since 1990)
▪	Number of positive lymph nodes		
**Additional data collection from medical charts**			
**Patient characteristics**		**Pregnancy**	
▪	Height	▪	Date
▪	Weight	▪	Duration
▪	Reproductive factors	▪	Complications
▪	Family history of breast cancer	▪	Abortion/miscarriage
▪	Comorbidities	▪	Delivery
▪	Fertility preservation	▪	Child health status
**Tumour characteristics**		▪	Child bearing
▪	Delay between symptoms and diagnosis	▪	Breast cancer status
▪	Method of detection		at time of pregnancy
▪	Diagnosis assessment	**Treatment**	
▪	Histological subtype	▪	Date
▪	Associated *in situ* carcinoma	▪	Type
▪	Multifocality	▪	Dose
▪	Estrogen receptor status (before 1995)	▪	Interruption for adverse effects
▪	Progesterone receptor status (before 1995)	▪	Patient refusal
▪	HER2 overexpression status (before 2003)	▪	Quality of treatment
▪	Ki-67 score (since 1997)	▪	Reason for delayed use
▪	Surgical margin	▪	Preservation of fertility
▪	Tumour necrosis	▪	Breast reconstruction
▪	Immunological reaction	**Others**	
	of peritumoral tissue	▪	Genetic counselling
			BRCA testing and result
▪	Blood vessel invasion	▪	Tumour Board
▪	Lymphatic vessel invasion		
**Outcomes**			
▪	Loco-regional recurrences	▪	Site
▪	Distant recurrences	▪	Pathology
▪	Dates	▪	Treatments
**Additional data collection from Cantonal Office of Population**			
	**Offspring of breast cancer patients born before and after breast cancer diagnosis**		
▪	Date of birth	▪	Gender
**Additional data collection from linkage with the Oncogenetic and Cancer Prevention Unit**			
▪	Genetic counselling		
▪	BRCA testing and result		

### Follow up

Patient follow-up included the recording of recurrences (loco-regional and distant), secondary malignancies, migration and vital status with the exact date of each event. The date of last follow-up was 31 December 2015.

Collection of data on second cancers, migration and vital status was done through the usual recording process performed at the Geneva Cancer Registry. The same procedure that was used to record a first primary cancer was used to register a second cancer for the same individual. Each year the OCP provides the Geneva Cancer Registry with information on migration and vital status of the Cantonal population.

Information on recurrences is not routinely recorded at the Geneva Cancer Registry. The collection of those data was performed retrospectively from medical charts. Patients who were still alive at the end of follow-up or who died of causes other than breast cancer, and for whom no information on recurrence was found through these strategies, were considered as relapse-free.

## Findings to date

The mean age at diagnosis of the 1,586 women in the cohort was 39.9 years (range, 22–45). Breast cancer was diagnosed before the age of 35 years in 225 women (14.2%), between 35 and 39 years of age in 368 women (23.2%) and between 40 and 45 years of age in 993 women (62.6%). Using data on Ki-67 or grade of the tumour, HER2 expression and estrogen and progesterone receptors, most of the patients were diagnosed with luminal A or luminal B molecular subtypes (32.8 and 37.5%, respectively). Triple negative breast cancers represented 10.4% of the tumours. Patients were more often diagnosed with Stage I or II tumours (75.2%), and positive estrogen (74.8%) and progesterone (67.5%) receptors (Table 2).

**Table 2:** Main individual and tumour characteristics for young women diagnosed with breast cancer in Geneva, Switzerland, 1970–2012.

**Age class (years)**				**Comorbidities**			
	<35	225	14.2		No	1’025	64.6
	35–39	368	23.2		Yes	108	6.8
	40–45	993	62.6		Missing	453	28.6
	Total	1’586	100		Total	1’586	100
**Period of diagnosis**				**TNM stage**			
	1970–1979	296	18.7		Stage I	504	31.8
	1980–1989	333	21		Stage II	689	43.4
	1990–1999	348	21.9		Stage III	130	8.2
	2000–2009	467	29.4		Missing	263	16.6
	2010–2012	142	9		Total	1’586	100
	Total	1’586	100	**Grade**			
**Social class**					1	192	17.3
	High	252	15.9		2	498	45
	Medium	684	43.1		3	343	31
	Low	510	32.2		Missing	74	6.7
	Missing	140	8.8		Total	1’107	100
	Total	1’586	100	**Progesterone receptors**			
**Nationality**					Negative	177	22.3
	Switzerland	1000	63.1		Positive	593	74.8
	Europe	462	29.1		Missing	23	2.9
	Asia, Africa & Oceania	81	5.1		Total	793	100
	America	43	2.7	**Estrogen receptors**			
	Total	1’586	100		Negative	235	29.6
**Birth place**					Positive	535	67.5
	Switzerland	728	45.9		Missing	23	2.9
	Europe	616	38.8		Total	793	100
	Asia, Africa & Oceania	161	10.2	**Ki-67 (Mib1)**			
	America	81	5.1		Negative	252	41.4
	Total	1’586	100		Positive	258	42.4
**Body Mass Index (kg/m**^**2**^**)**					Missing	99	16.3
	<20	111	7		Total	609	100
	20–24	282	17.8	**HER2 overexpression**			
	25–29	81	5.1		No	391	67.9
	30+	46	2.9		Yes	122	21.2
	Missing	1’066	67.2		Missing	63	10.9
	Total	1’586	100		Total	576	100
**Origin of diagnosis**				**Molecular subtypes**			
	Clinical examination	689	43.4		Luminal A	189	32.8
	Fortuitous	50	3.2		Luminal B	216	37.5
	Opportunistic screening	260	16.4		HER2-enriched	33	5.7
	Self-examination	474	29.9		Triple negative	60	10.4
	Missing	113	7.1		Missing	78	13.5
	Total	1’586	100		Total	576	100
**Oncogentic consultation**							
	No	1304	82.3				
	Yes	282	17.8				
	*Tested*	*185*	*65*.*6*				
	*Not tested*	*97*	*34*.*4*				
	Total	1586	100				

Almost all women underwent surgery, with breast-conserving surgery in 62% of the patients and mastectomy in 36%. Radiotherapy, chemotherapy and hormonotherapy were administered to 70%, 55% and 36% of the 1,586 patients, respectively.

The median follow-up period was 10.2 years (range, 0.01–45.8 years). During the study period, 16.7% of the women (N = 265) developed local recurrences and 25.4% (N = 403) developed distant metastases; the majority did not have a recurrence (N = 1,051, 66.3%). In terms of mortality, 474 women (29.9%) died during the study period, 347 (73.2%) from breast cancer. A considerable number of women were lost to follow-up during the study period (19.5%). When patients who were lost to follow-up were compared to those who remained, no statistical difference was found in terms of individual (age and social class status) or tumour characteristics (grade and stage of the disease) (Table 3). However, women lost to follow-up were more likely to have a non- Swiss nationality (Table 3). Breast cancer was diagnosed during pregnancy in 1.3% of patients (N = 20) and within one year following childbirth in 3.3% of patients (N = 53).

**Table 3:** Comparison between patients who were lost to follow up versus those not lost to follow-up.

	Cohort		Lost to follow-up		Total		P-value for chi^2^ test
	N	%	N	%	N	%	
**Age class**							0.060
<35	175	77.8	50	22.2	225	100	
35–39	284	77.2	84	22.8	368	100	
40–45	817	82.3	176	17.7	993	100	
**Birth place**							0.000
Switzerland	631	86.7	97	13.3	728	100	
Europe	473	76.8	143	23.2	616	100	
Asia, Africa, Oceania	119	73.9	42	26.1	161	100	
America	53	65.4	28	34.6	81	100	
**Nationality**							0.000
Switzerland	848	84.8	152	15.2	1000	100	
Europe	343	74.2	119	25.8	462	100	
Asia, Africa, Oceania	63	77.8	18	22.2	81	100	
America	22	51.2	21	48.8	43	100	
**Social class**							0.298
High	204	81	48	19	252	100	
Medium	548	80.1	136	19.9	684	100	
Low	419	82.2	91	17.8	510	100	
Unknown	105	75	35	25	140	100	
**Stage**							0.707
I	409	79	109	21	518	100	
II	616	81.6	139	18.4	755	100	
III	131	79.9	33	20.1	164	100	
Unknown	120	80.5	29	19.5	149	100	

**Fig 1:**
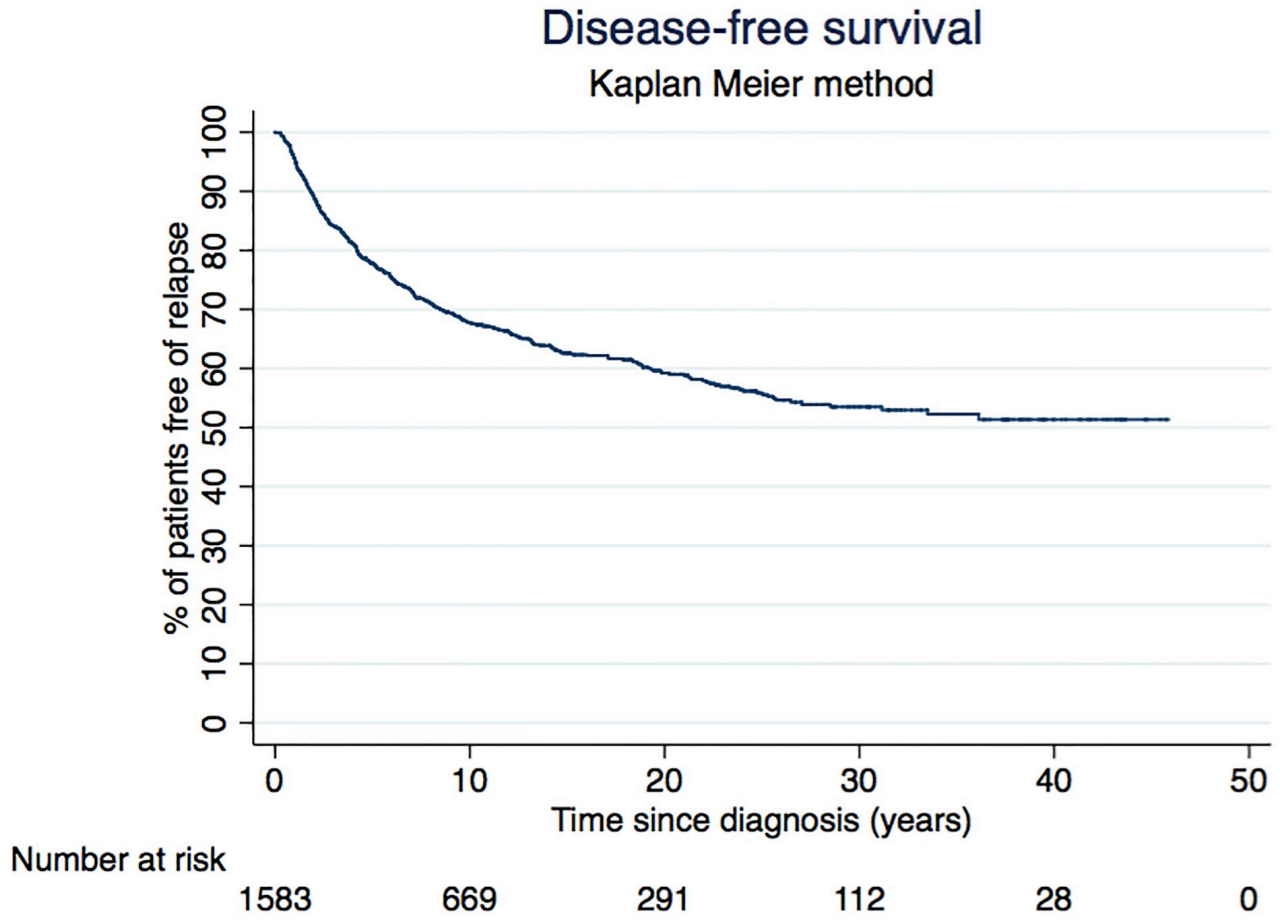
Disease free survival for breast cancer patients diagnosed between 1970 and 2012 in Geneva.

The time between diagnosis and first relapse (loco-regional or distant) or date of last follow-up was used to calculate the disease-free survival (DFS) by Kaplan–Meier method. Thirteen patients were excluded from this analysis because of missing dates. The total number of relapses considered was therefore 522. Overall, the DFS rate at 10 years was 0.68 (95% CI: 0.66–0.71). The DFS rate decreased to 0.60 (95% CI: 0.57–0.63) at 20 years and remained stable thereafter (Fig 1).

### Strengths and limitations

This is the first study to investigate the long-term health outcomes in a population-based cohort of young breast cancer patients with complete information on patient, tumour and treatment characteristics. The strengths of this study include the high quality of data available at the Geneva Cancer Registry and the capacity to merge it with additional databases. This enables both cross-validations and the inclusion of supplementary variables.

Information on recurrence was collected retrospectively during the study by re-opening the patients’ medical charts rather than recorded at the time of occurrence. We are confident, however, that all available information was captured due to the strong, high quality network of experts that operates in this geographically restricted area. Furthermore, the assumption that “no information” means “no relapse” was tested and validated for a random sample of 30 cases for which an active search was performed using all available sources including direct contact with patients’ physicians.

Germline *BRCA1/BRCA2* pathogenic variants are an important factor in studying young women with breast cancer. Second primary breast cancers are more frequent in carriers of *BRCA1*/*BRCA2* mutations and this higher frequency drives early genetic testing to inform surgical decision-making, even though recent studies have questioned the role of *BRCA1/BRCA2* mutations in breast cancer survival [16]. In our population-based cohort not all patients had genotyping for pathogenic *BRCA1*/*BRCA2* mutations. However, information obtained by linking the cohort dataset with that of the onco-genetic counselling unit enables the evaluation of potential biases.

The main shortcoming for our cohort is the proportion of patients who were lost to follow-up (19.5%). The comparison of these patients with those who remained in the risk set revealead as a sole difference the nationality, being women lost to follow-up more often of a non-Swiss nationality, which corresponds to the general structure of the Geneva population with more than 40% of the residents coming from outside the Canton. Finally, although a patient who is lost to follow-up cannot present as a death event, such patients could still present as a loco-regional and/or a distant recurrence.

## Conclusions

Breast cancer in young women remains poorly understood. Many uncertainties exist in terms of prognostic factors, treatment, recurrence and mortality. Uncertainties are even greater in terms of the risks posed by pregnancy during and after breast cancer in this population. This project would contribute to the accumulating knowledge on factors that influence the risk of local and distant recurrence and the probability of survival in young women with breast cancer. Enhancing understanding of the risks associated with pregnancy during and after breast cancer in this age group is internationally recognized as a research priority. The creation of this population-based dataset on breast cancer in young women is envisioned to be the first step towards the constitution of a Swiss Observatory of young breast cancer patients through which the burden of disease, incidence and mortality trends, quality of care, prognostic factors and outcomes can be monitored on an ongoing basis.
